# Influence factor and mechanism of FLASH effect

**DOI:** 10.3389/fonc.2025.1669228

**Published:** 2025-09-17

**Authors:** Tingyu Feng, Tianyang He, Wentao Ye, Lisha Xiang

**Affiliations:** 1 Division of Thoracic Tumor Multimodality Treatment and Department of Medical Oncology, Department of Radiation Oncology, Cancer Center, West China Hospital, Sichuan University, Chengdu, China; 2 West China School of Medicine, West China Hospital, Sichuan University, Chengdu, China

**Keywords:** ultra-high dose-rate, FLASH radiotherapy, mechanism, influencing factor, FLASH effect

## Abstract

FLASH radiotherapy (FLASH-RT) represents an innovative treatment modality utilizing ultra-high dose-rate irradiation (>40 Gy/s). The FLASH effect, induced by FLASH-RT, is characterized by the selective radioprotective effect of normal tissue while preserving tumor control efficacy. Currently, FLASH effect has been confirmed in many preclinical studies and clinical studies. However, the mechanism and the influencing factors of FLASH effect remain ambiguous. This review systematically summarizes current understanding of the mechanism and influencing factors of FLASH effect, providing theoretical basis for the future study and application of FLASH-RT.

## 1 Introduction

Cancer has become a leading cause of human death in modern society (1). In 2024, 611720 people died from cancer in America (2). As one of the most important and effective treatment for cancer, almost 50% of all cancer patients received radiotherapy during the course the disease (3). However, radiotherapy also caused early or late toxicities in organs at risk (OARs) surrounding the tumor, which limit the patient’s survival and quality of life (4). Although modern precision radiotherapy techniques (e.g., 4D-CT (5), VMAT (6), TOMO (7)) have achieved improved normal tissue complication control through image-guided targeting and algorithm-optimized dose distribution, such protection is still limited by the physical dosimetry of the radiation itself. In conventional radiotherapy (CONV-RT), precision techniques can only minimize the radiation exposure of normal tissues, while reducing the radiation-induced damage after exposure is impossible. To make matters worse, due to the inter-patient variability and uncertainties caused by respiratory motion (8), even the most advanced intensity-modulated techniques cannot completely avoid radiation exposure to normal tissues.

Recent studies have shown that radiation delivered with an ultra-high dose rate (≥40 Gy/s, UHDR) can significantly reduce the damage to OARs without compromising the anti-tumor effect (9). This phenomenon is called “FLASH effect”, which can essentially mitigate the toxicity of radiotherapy. FLASH effect was first discovered by Dewey and Boag in 1959, although they only demonstrated that *Serratia marcescens* under UHDR irradiation presented lower radiosensitivity (10). The FLASH effect was subsequently confirmed in mammalian cells (11), but its connection with cancer treatment was not established until 2014. In 2014, Vincent Favaudon’s group used FLASH-radiotherapy (FLASH-RT) to treat lung cancer in mice and found that compared with conventional dose rate (CONV) irradiation (0.03 Gy/s), FLASH-RT (60 Gy/s) resulted in reduced damage in heathy tissue while maintaining tumor control (9). After that, diverse preclinical studies have been conducted, confirmed FLASH effect in zebrafish (12), canine-cancer patients (13), cat-cancer patients and mini-pig (14).

In 2019, the first FLASH-RT clinical trial was reported, which demonstrated the protective effect of electron FLASH-RT on normal skin tissue (15). The consistent tumor control efficacy was further supported by the subsequent two-year follow-up survey (16). The first proton FLASH-RT clinical study was conducted on patients with extremity bone metastases, which also confirmed that FLASH-RT could achieve equivalent therapeutic outcomes with CONV-RT (17). However, since the irradiated area of this experiment was located on the limbs, distant from the radiation-sensitive organs, the protective effect of reducing normal tissue damage was not significantly demonstrated. A similar trial will soon be initiated for thoracic metastases patients (18). More clinical trials, like the one related to cutaneous squamous cell carcinoma (19) are still undergoing. Generally, those prospective studies have validated the clinical feasibility and safety of FLASH-RT based on their promising data.

In the past, the only way to limit the radiotherapy toxicity was to widen the gap between the optimal tumor-controlling dose and the minimal OARs-causing dose (20), or to reduce the irradiated volume of normal tissues by using precision radiotherapy. However, the discovery of the FLASH effect gives out another possible solution. By altering the intrinsic dose-rate characteristics of radiation, FLASH-RT fundamentally reduces the radiation-induced damage of normal tissues while significantly decreasing the time required for each treatment session. This approach significantly increases the deliverable dose per fraction, which enhances the tumor killing effect and reduces the treatment duration. Furthermore, as a treatment method derived from the fundamental radiation parameter modification, FLASH-RT can seamlessly integrate with intraoperative radiotherapy, precision radiotherapy, immunotherapy and other therapeutic approaches, demonstrating remarkably broad developmental potential.

Although the FLASH effect has already been confirmed in electron beams (9), protons beams (21), X-rays (22, 23) and heavy ions (24), the mechanism underlying the FLASH effect occurrence remains poorly understood. Besides, the occasionally occurred negative results also suggested the highly complex and diverse influencing factors associated with the FLASH effect (25), which seems ambiguous currently as well. To achieve the FLASH effect safely and consistently, it is important to elucidate the underlying mechanism and control the relative effecting factors. This will enable precise modulation of the FLASH molecular pathways and facilitate the establishment of international standards for FLASH treatment protocols. Furthermore, since induction of the FLASH effect requires precise control of both dose rate and temporal parameters, the development of high-precision dosimetry systems and beam delivery technologies has also become equally imperative. Tackling these challenges will be the focus of radiotherapy research for the foreseeable future.

This review summarizes the research progress on the mechanism and influencing factors of the FLASH effect and provided a theoretical basis for the future studies and clinical applications of FLASH-RT.

## 2 Factors influencing FLASH effect

### 2.1 Biological factors

#### 2.1.1 Age and oxygenation

The manifestation of FLASH effect in practical irradiation is often influenced by multiple factors, with biological factors including the aging status of irradiated tissues, oxygen content, and tissue type (**Table 1**). Regarding aging status, a mouse study has shown that 17 Gy of FLASH-RT failed to elicit the FLASH effect in telomerase-knocked mice, which revealed that the advanced age and decreased telomerase activity of the irradiated subject may impede the manifestation of the FLASH effect (26). However, to date, further research of this phenomenon remains limited, with the underlying mechanisms remaining elusive. Subsequent studies focusing on this phenomenon are still warranted.

**Table 1:** Summary of biological and physical factors influencing the FLASH effect.

Influencing factor		Year	Author	Dose (Gy)	Dose rate (Gy/s)		Radiation source	Endpoint
					CONV-RT	FLASH-RT		
Age		2020	Charles F (26)	17	0.03	60	Electron	FLASH effect absented in mice without telomerase activity.
Oxygenation		2024	Jacob P S (27)	19.8	0.16	25~1170	Electron	FLASH effect was restricted in low oxygen level.
		2022	Pankaj C (29)	0.5~4	0.067	2×10^9	Proton	Vitro study showed DNA DSB residues enhance under hypoxic conditions.
		2023	Hai Siong Tan (30)	Mathematical modeling and computation				Physical model showing hypoxic environment limited FLASH effect.
Organ	Intestine	2021	Jia-Ling Ruan (34)	7.5~12.5	0.25	106	Electron	FLASH-RT enhanced crypt survival and minimized microbiome changes in mice.
		2023	Anet Valdés Z (33)	11~17	0.17	185~225	Electron	Identical parameters induced robust, reproducible FLASH effect in intestine.
		2020	Eric S (21)	15	0.9	78	Proton	FLASH-RT restrict the death of intestinal crypts.
				18				FLASH-RT limited the intestinal fibrosis level.
		2022	Hongyu Zhu (32)	10~15	NA	>150	X-ray	FLASH-RT increased the survival rate and body weight recovering of mice.	
		2024	Tristan L Lim (35)	14	0.82	125.3	Proton	FLASH-RT enhances progenitor proliferation and tissue regeneration.	
	Skin	2021	Anastsia V (36)	30, 45	0.39~0.65	69~124	Proton	FLASH-RT mitigates apoptotic signaling transformation.	
		2021	Brita Singers S (37)	23.2~39	0.35~0.40	65~92	Proton	FLASH-RT requires 44-58% higher doses than CONV-RT for equivalent effects.	
		2023	Qixian Zhang (39)	25~30	0.40	130	Proton	FLASH-RT reduce skin injury; oxygen abolishes dose-rate variance.	
		2024	Per Rugaard P (40)	39.3	0.37	80	Proton	OER dose quantifies FLASH effects on murine skin toxicity.	
		2020	Luis A Soto (38)	30~40	0.074	207	Electron	FLASH-RT induced milder skin ulcers and prolonged median survival.	
	Lung	2014	Vincent Fn (9)	17	0.03	60	Electron	FLASH-RT prevent lung fibrosis and spared smooth muscles.
		2020	Young-Eun K (42)	15	0.06	352.1	Electron	FLASH-RT elicit less myosin light chain, changing tumor microenvironment.
	Brain	2022	Ivana Dokic (46)	~10	0.17	120	Proton	FLASH-IR preserves microvascular architecture, attenuates inflammation.	
		2024	Ivana Dokic (47)	10	0.2	250	Helium	FLASH-RT reduces brain damage, preserves neurovascular endothelium.	
		2019	Danielle A (44)	30	0.13	200~300	Electron	Fewer hippocampal dendritic loss and neuroinflammation induce FLASH effect.	
		2025	Jeannette J (48)	10	0.1	5.5×10^6	Electron	FLASH-RT restricts hippocampal atrophy and microstructural alterations.	
		2024	Olivia G (49)	10	0.1	225	Electron	FLASH-RT helps recovery of immature neurons.	

CONV-RT, conventional radiotherapy (40< Gy/s); FLASH-RT, ultra-high dose rate radiotherapy (≥40 Gy/s); OER, oxygen enhancement ratio.

Similar reduction in FLASH effect can also be found in hypoxic tissues (27). Both *in vitro* studies (28, 29) and physical models (30) have demonstrated that under hypoxic conditions, FLASH effect is inhibited and cannot exert significant radioprotective effect after irradiation.

#### 2.1.2 Tissue characteristic

Moreover, among different tissues, FLASH effect also demonstrates with varying intensity levels and distinct manifestation patterns. This variability is primarily related to the diverse oxygenation levels and mitotic activity in different organs and tissues, which lead to their differential radiosensitivity. Generally, tissues with higher radiosensitivity are more susceptible to radiation damage, which may make the protective effect of FLASH effect more pronounced. Consequently, this leads to significant variations in the conventional irradiation parameters required to achieve the FLASH effect across distinct tissues. Meanwhile, the manifestation of FLASH effect also varies according to the type of irradiated tissue or organ (**Table 2**).

**Table 2:** Summary of tissue-specific characteristics and radioprotection.

Tissue	Characteristic			Dose (Gy)	Radioprotection observed
	Mitotic activity	Position	Radiation damage		
Intestine	High	*In vivo*	Targets rapidly proliferating cells	10-15	Preservation of cellular proliferative capacity (35)
Skin	Moderate	Surface		20-40	Maintain proliferative capacity (36); Oxygen partial pressure-dependent effect (39)
Lung	Low	*In vivo*	Inflammation and fibrosis	15-18	Suppression of pro-inflammatory genes (26); Reduction of fibrotic changes (9)
Brain	Very low	*In vivo*	Neuronal cell damage	10	Protection of neuronal activity and structural integrity (44, 49)

Typically, the lungs, intestines, brain, and skin are the most vulnerable organs in radiation therapy, making the FLASH sparing effect of great significance. In this section, we take these four tissues as examples to analyze how tissue-specific characteristics influence the FLASH effect.

##### 2.1.2.1 Intestine

Tissues with highly proliferating cells (e.g., intestine/skin) exhibit greater radiosensitivity and are more prone to acute radiation injury, whereas terminally differentiated tissues (e.g., brain/lung) predominantly manifest delayed damage. Generally, rapidly dividing tissues demonstrate more pronounced FLASH effects at lower doses of irradiation. Among the four tissue types, intestinal epithelial cells exhibit the most active proliferation, with their crypt stem cells capable of continuous division and complete renewal of the intestinal epithelium every 3–5 days. This characteristic enables the intestinal epithelium to demonstrate consistent FLASH effects at relatively low radiation doses (10–15 Gy). With a total abdominal FLASH-RT in mice, Karen et al. certified that after 16 Gy of irradiation, CONV-irradiated mice experienced continuous weight loss and died 10 days post-irradiation (dpi), while FLASH-irradiated mice recovered their body weight within days and survived (31). Similar results have been found by other researchers, confirming that the FLASH effect can be consistently reproduced in gut (21, 32, 33).

However, the high radiosensitivity of intestinal tissue also suggests that with higher radiation doses, the protective FLASH effect in intestinal tissue may be readily overshadowed by more severe radiation damage, thereby imposing stricter requirements on dose parameters and beam characteristics for demonstrating FLASH effects in the small intestine. For example, for Zhang et al., following a partial-gut FLASH-RT using protons, neither intestinal tissue nor circulating lymphocytes were spared, indicating that the FLASH effect did not occur (25). Since other studies often employed 10–15 Gy of electron beams for irradiation, this difference may result from the relatively higher radiation doses employed in this experiment (14~18 Gy), combined with more severe damage caused by proton irradiation.

Microstructural investigations revealed that the radioprotective effect of FLASH-RT in gastrointestinal tract primarily manifests as reduced mortality of crypt base columnar cells (CBCs), essentially preserving their proliferative capacity. Researches showed that after 12.5 Gy of FLASH-RT, mice exhibited fewer changes in their gut microbiome composition and higher survival rate of proliferating cells in intestinal crypts, which contribute to the preservation of gut function (34). Further studies supported this finding and demonstrated that FLASH-RT also promoted greater proliferation of epithelial progenitor cells, leading to a better regeneration after irradiation (35).

##### 2.1.2.2 Skin

The proliferation of skin basal cells occurs at a relatively slower rate, with basal keratinocytes renewing the epidermal layer every 14–28 days. Consequently, studies of the FLASH effect in cutaneous tissue typically utilize higher radiation doses (20–40 Gy) compared to those used for intestinal tissue. The FLASH effect of mammal cutaneous tissue was first discovered in cat and mini-pig in 2018 (14). The first human experiment then revealed that FLASH-RT demonstrated both safety and feasibility, with significant protective effects on skin tissues and robust antitumor efficacy (15). For skin, radiation damage primarily manifests as erythema, desquamation, and suppuration triggered by basal cell death. In contrast, the FLASH effect is chiefly characterized by mitigating radiation-induced follicular atrophy, stem cell depletion, apoptotic signaling activation, inflammation, and ulceration in the irradiated area (36–38).

However, unlike other tissues, as the outermost organ exposed to the external environment, FLASH effect in cutaneous tissue is profoundly influenced by the local oxygenation. In a study conducted by Qixian Zhang et al., although FLASH-RT demonstrated a 15% reduced skin contraction and sustained protective effects, both extra oxygen supplementation and oxygen reduction could abolish these dose-rate-dependent variations (39). Furthermore, Per Rugaard Poulsen et al. even proposed that the oxygen enhancement ratio-weighted dose could accurately describe the acute cutaneous toxicity changes in mice, highlighting the FLASH effect’s profound dependence on oxygen partial pressure in skin tissue (40).

##### 2.1.2.3 Lung

For tissues with low proliferative activity, although they exhibit lower radiosensitivity, their capacity for post-radiation self-repair is markedly weaker. Furthermore, these tissues - particularly critical organs like the lungs and brain – often play essential roles in maintaining normal physiological functions. Consequently, although the FLASH effect provides relatively modest radioprotection for such tissues, its clinical significance is exceptionally high. For these tissues, FLASH effect is strongly influenced by the distinct mechanisms of radiation damage formation.

In pulmonary tissue, radiation-induced damage primarily manifests as tissue fibrosis triggered by post-radiation inflammation and edema caused by altered vascular permeability. The protective effects of FLASH radiotherapy are likewise mainly demonstrated in these two aspects. FLASH effect in lung was first identified in 2014, when study confirmed the comparable tumor control along with the restricted pulmonary fibrosis following FLASH-RT in mice (9). In this experiment, FLASH-irradiated mice exhibited higher survival rates during long-term observation, with no significant complications observed. In contrast, CONV-irradiated mice developed severe pneumonitis, inflammatory cell infiltration, and pre-fibrotic lesions, resulting in decreased survival rates. Subsequent studies have further delineated the radioprotection effect in pulmonary tissues at cellular and molecular levels. Fouillade et al. reported reduced expression of pro-inflammatory genes, better preservation of progenitor pool, and decreased cellular senescence after FLASH-RT, leading to a higher lung regeneration capacity, which indicating a better prognosis outcome (26). In pulmonary fibroblast cells, they also observed fewer double-strand break foci, although another *in vitro* study reported no significant difference in fibroblast survival between FLASH-RT and CONV-RT (41). Furthermore, research of microenvironmental alterations revealed that FLASH-RT also suppressed the activation of myosin light chain in vascular endothelium, consequently reducing the capillary constriction and promoting the infiltration of immune cells (42).

##### 2.1.2.4 Brain

In brain tissue, radiation-induced damage primarily manifests as cognitive impairments associated with neuronal cell injury and hippocampal alterations. In that case, the protective effects of FLASH radiotherapy are predominantly focused on preserving neural architecture and maintaining neuronal viability (22). In brain, FLASH effect was first demonstrated by Pierre Montay-Gruel et al. in 2017, with a convincing result showing that in whole brain irradiation, 10 Gy of CONV-RT (0.1 Gy/s) could totally damage the spatial memory of mice, while same dose of FLASH-RT (100 Gy/s) could preserve the memory of mice for two months (43). Subsequent studies uncovered that this neuroprotection was mediated through the reduced neuroinflammation and hippocampal dendritic spine loss after irradiation (44), which were associated with the fewer toxic hydrogen peroxide production after FLASH-RT (45). Further study of Ivana Dokic et al. not only supported the attenuation of microglia and macrophage induced inflammation, but also uncovered the microvascular protection effect mediated by FLASH-RT (46, 47). A recent study utilizing ex vivo high-resolution brain magnetic resonance imaging demonstrated that FLASH-RT prevents hippocampal intensity from reduction, with concurrently conducted analyses showing negligible alterations in regional diffusion metrics across FLASH-irradiated mice (48). Another investigation revealed that the FLASH effect enhances post-irradiation recovery of immature neuronal cells, highlighting its specific neuroprotective effects on neuronal bioactivity (49).

### 2.2 Physical factors

#### 2.2.1 Dose and dose rate

In terms of physical factors, the FLASH effect is predominantly influenced by radiation dose, dose rate, pulse structure characteristics, irradiation field, as well as the type of radiation employed (**Table 3**). For dose rate, research on zebrafish embryo showed that higher dose rate (>40 Gy/s) and shorter irradiation time can significantly reduce the incidence of malformations (50). Further studies proved this conclusion and pointed out that the radioprotective efficacy of FLASH effect also increased with higher irradiation dose (51, 52). However, this increase tends to reach saturation around 50 Gy (52) and the whole protection capability will be almost negligible below 2 Gy (59). These findings not only proved that the flash effect is determined by both dose and dose rate parameters, but also provided a guidance on fractionation regimens for FLASH-RT. Existing experiments have demonstrated that standard fractionated FLASH-RT (2 Gy/fraction) still present FLASH effect (53, 54), although the intensity is likely to be reduced (55). The specific mechanism underlying this reduction is not yet clear, but it can typically be mitigated by increasing the dose per fraction (51).

**Table 3:** Evidence of physical factors influencing the FLASH effect.

Influencing factor		Year	Author	Dose (Gy)	Dose rate (Gy/s)		Radiation source	Endpoint
					CONV-RT	FLASH-RT		
Dose Rate		2022	Leonhard K (50)	30~33	0.12	177.2~2.5×10^5	Proton, Electron	FLASH effect enhances with dose-rate, reduces with irradiation time.
Dose		2022	Till T B (51)	11~14	0.3	1440	Electron	Higher irradiation dose enhances FLASH effect.
		2024	Felix H (52)	15~50	0.1	0.9×10^5	Electron	Increase of FLASH effect saturated in 50 Gy.
				20–95	0.33	240	Proton	
		2020	Jian-Yue J (59)	2~50	0.0017~0.083	40	Electron	FLASH effect peaks at 30~50 Gy, vanishes below 2 Gy.
		2023	Charles L L (53)	10×3	0.09	1.6×10^6	Electron	30 Gy FLASH achieves normal Long-term potentiation like controls
		2023	Yuling D (54)	10×2	0.03	200	Electron	2 Gy fractions elicit FLASH effect.
		2024	Brita S S (55)	39.3	0.7	80	Proton	Dose fractionation weakens FLASH effect.
Pulse Structure		2021	Jia-Ling (34)	7.5~12.5	0.25	106	Electron	Pulse number and interval time increase, sparing effect reduce.
		2024	Kevin L (56)	12~14	0.2, 0.3	150, 230	Proton	Dose per pulse increase, pulse width shrinks, FLASH effect enhance.
Irradiation Area		2022	Carla R B (57)	31	0.1~0.15	1500	Electron	FLASH effect increases when irradiation field shrink.
Radiation Type	Electron Beam	2024	Kevin L (58)	10×3	0.09	1.6×10^6	Electron	eFLASH have better sparing effect of normal tissue.
		2023	William T (61)	8~30	0.1	115~660	Electron	eFLASH better restricts H_2_O_2_ production and O_2_ consumption than pFLASH.
				15~30	10	80	Proton	
	Proton Beam	2022	Lorea I (60)	25	4	257	Proton	Protection of circulating immune cell vanished in pFLASH.
		2022	Houda K (62)	15~95	0.1	0.9×10^5	Electron	pFLASH showed lower restriction of H_2_O_2_ generation than eFLASH.
					0.33, 0.5	240, 600	Proton	
	X-ray	2022	Xiaolin S (63)	13	0.03	110~120	X-ray	X-ray FLASH-RT protect the irradiated intestinal crypts.
		2019	Feng G (23)	15~30	0.1	1200	X-ray	X-ray FLASH-RT reduced mortality in irradiated mice.
	Heavy Ion	2022	Walter T (64)	18	0.3	100	Carbon-ion	FLASH-RT reduced the structural changes in muscle.
		2024	Walter T (24)	20	0.33	>100	Carbon-ion	FLASH-RT irradiation reduces collagen deposition in muscle.
		2022	Mutsumi T (65)	1~3	8~13	96~195	Carbon-ion	No FLASH effect was observed in human lung fibroblasts.

CONV-RT, conventional radiotherapy (40< Gy/s); FLASH-RT, ultra-high dose rate radiotherapy (≥40 Gy/s); eFLASH, electron FLASH irradiation; pFLASH, proton FLASH irradiation.

#### 2.2.2 Pulse structure and irradiation area

Additionally, pulse structure also serves an important role in influencing FLASH sparing effect. On the one hand, when increasing the pulse number or lengthen the interval time between two pulses, the sparing effect of FLASH effect will be suppressed (34). On the other hand, it is possible to enlarge the dose per pulse or shrink the pulse width to reach a better protection effect (56). Moreover, shrinking the irradiation field has been shown to enhance the FLASH effect. Study on skin of mini-pig revealed that when utilizing larger irradiation field (8×8 cm²), even FLASH-RT (150 Gy/s) would result in severe late toxicity, indicating that increased treatment volume significantly compromises the radioprotective FLASH effect (57).

#### 2.2.3 Type of radiation

Current clinical radiotherapy predominantly utilizes three radiation modalities: electron beams, proton beams, X-rays and heavy ion. Extensive studies have confirmed the FLASH effect in all four types of radiations. Recent studies have also shown that the magnitude of FLASH effect also exhibits radiation-type dependence, attributable to their inherent differences in physical characteristics (**Table 3**).

An abdominal irradiation experiment in mice showed that with identical dose and dose rate, no significant difference could be found between conventional electron irradiation (eCONV) and proton irradiation (pCONV), while a significant difference was observed between electron FLASH irradiation (eFLASH) and proton FLASH irradiation (pFLASH). The number of regenerating crypts in mice treated with eFLASH was 2–5 times higher than those treated with pFLASH, so do the survival rates, which indicated the superior normal tissue protection of eFLASH (58). This may result from the distinct linear energy transfer (LET) characteristics between eFLASH and pFLASH, with proton beams in our experiments demonstrating LET values of 0.9-1.1 keV/μm (shoot-through region) or 1.25-2.8 keV/μm (spread-out Bragg peak region), while electron beams consistently showed lower LET ranges of 0.2-0.3 keV/μm. Similar conclusion was also made in studies of circulating immune cells, for which the sparing effect presented in eFLASH (59) and vanished in pFLASH (60). Subsequent mechanistic studies revealed that although both pFLASH and eFLASH showed a reduced reactive oxygen species (ROS) generation after FLASH-RT, eFLASH exhibited more distinctive advantages in other aspects. In terms of hydrogen peroxide (H_2_O_2_) production, pFLASH only leaded a reduction of 5%, whereas eFLASH resulted in a 69% reduction. For oxygen consumption, the change between the UHDR/CDR ratio was 22% for pFLASH and 43% for eFLASH, indicating a larger decrease in oxygen consumption of UHDR electronsirradiation (61). The greater reduction in H_2_O_2_ production by eFLASH was also demonstrated in the study of Houda et al. (62).

May attributed to the technical challenges in achieving UHDR high-energy X-rays, current research has only reported consistent FLASH effects with X-ray irradiation (23, 32, 63), while comparing between X-ray FLASH and eFLASH/pFLASH remains limited. Similar to X-ray FLASH, currently there are still no comparative studies evaluating the relative efficacy between heavy-ion FLASH and eFLASH/pFLASH modalities. However, although the studies of osteosarcoma mouse model have confirmed the existence of protective effects in carbon-ion FLASH-RT (24, 64), experiment on human cell yielded negative results, showing no FLASH effect was observed after 96–195 Gy/s carbon-ion irradiation (65).The emergence of negative results suggests the possibly less effective of heavy-ion radiation in inducing the FLASH effect. Meanwhile, since the positive results are limited to osteosarcoma models presently, their reproducibility and generalizability remain to be further validated. Compared to the eFLASH and pFLASH, research on heavy-ion FLASH remains significantly limited, and more experimental evidence is urgently needed to validate the capability of heavy-ion radiation to trigger the FLASH effect.

In future investigations, it is essential to obtain more comparative data among UHDR X-rays, heavy ion, electron and proton of the difference in FLASH effect inducing. These data will provide critical guidance for subsequent FLASH experimental design and radiation modality selection in applications of FLASH-RT.

In addition to the influence of radiation’s physical characteristics on inducing the FLASH effect, the radiobiological properties also significantly affect FLASH-RT’s clinical application. For example, electron beams provide cost-effective options but are limited to superficial tumors. By contrast, proton beams and heavy ions deliver superior depth-dose distribution (Bragg peak) yet require substantially greater infrastructure investment (66). Achieving UHDR with high-energy photons-based radiotherapy remains technically challenging despite its potential for balanced cost and penetration depth (23). These radiation-specific characteristic provide essential guidance for radiation modality selection of FLASH-RT implementation across various tumor types and anatomical sites.

Beyond all that factors mentioned above, there are still many uncharacterized determinants are modulating the FLASH effect, requiring systematic investigation. Further investigations into these factors will establish a solid foundation for mechanistic and translational research, which is of great clinical implications.

## 3 Mechanism of FLASH effect

### 3.1 Transient oxygen depletion hypothesis

The oxygen depletion mechanism is one of the most widely accepted mechanistic explanations (67). As early as 1980, the radioprotective effect of low oxygen concentration had already been established, and the Bohlen differential cell mortality between normoxic and hypoxic conditions was termed oxygen enhancement ratio (OER) (68). Therefore, once the FLASH effect was proposed, the radiation-induced transient hypoxia was immediately identified as one of the most possible underlying mechanisms (9).

**Figure 1:**
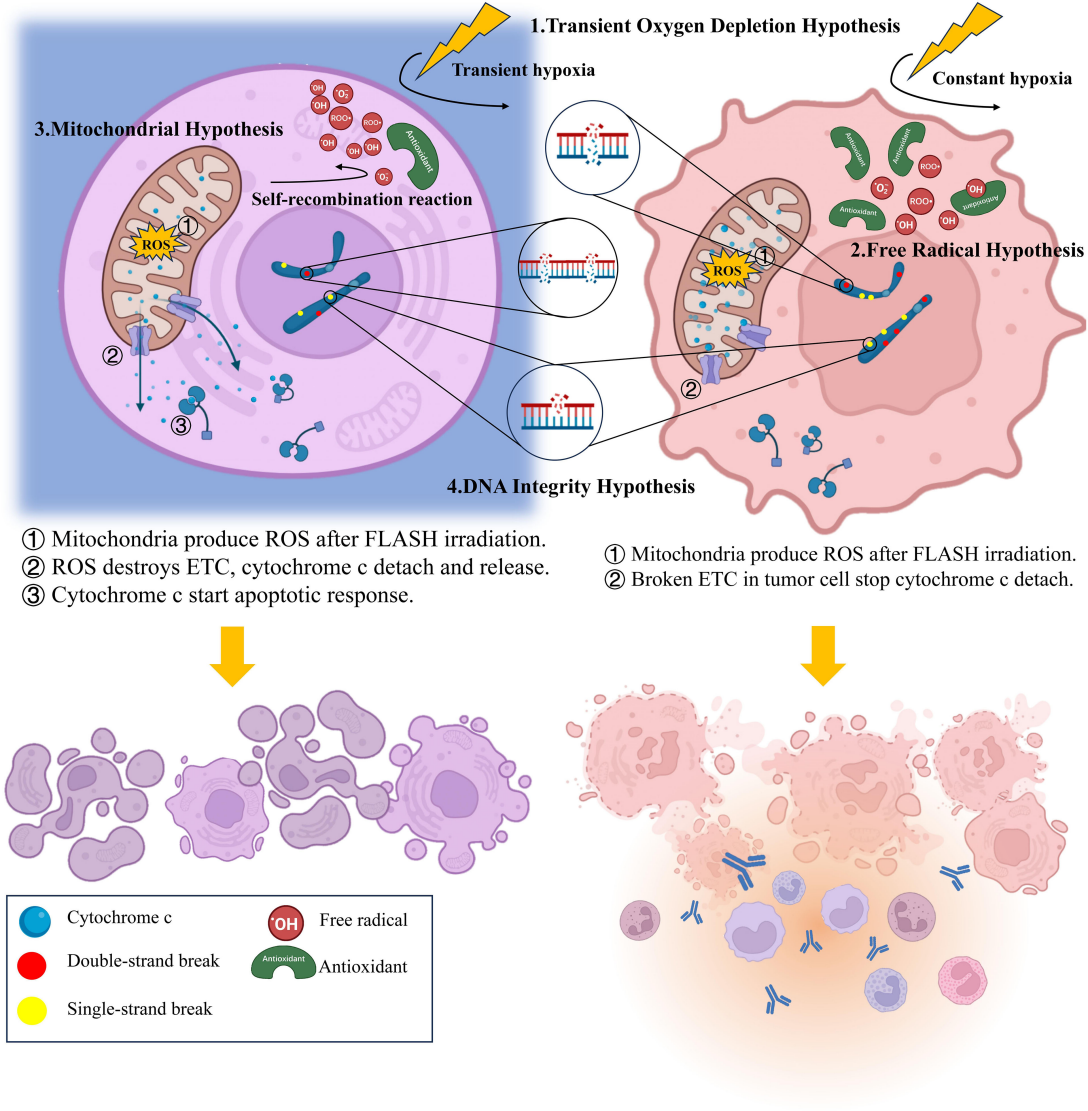
Summary of the biological mechanism of FLASH effect.

According to this hypothesis, large dose of radiation delivered within a short period can reacts violently with water, producing a large amount of reducing radicals within a second. The burst of reducing radicals immediately trapped all the intratissue oxygen, forming a transient hypoxia environment, and therefore protecting the irradiated tissue. This protection usually manifested as reduced ROS generation and decreased peroxide yield (45). In contrast, CONV-RT, due to its prolonged delivery time and lower dose-rate efficiency, often fails to counteract tissue reoxygenation during treatment, preventing the establishment of a hypoxic environment (69). Consequently, the slowly generated high flux of reducing radicals continuously reacts with available oxygen, producing substantial amounts of ROS, causing great damage to both tumor and normal tissue (70). Since the oxygen level of normal tissues usually much higher than that of tumor tissues, the oxygen depletion and protective effects in normal tissues are more pronounced, while the one in tumor tissues nearly invisible (**Figure 1**).

Supporting research revealed that the sparing effect of FLASH-RT is abolished under anoxic conditions, demonstrating the essential role of oxygen depletion in mediating the FLASH effect (71). Further studies demonstrated that FLASH-RT can also reduce the oxygen consumption *in vitro* condition, explaining the discovered FLASH effect under normoxic conditions (41). However, the weak correlation between the heavy ion FLASH effect and oxygen concentration suggests that in addition to oxygen depletion theory, there are still more complex mechanisms contribute to this phenomenon (72).

### 3.2 Free radical hypothesis

To fill the gap in oxygen depletion theory, Labarbe et al. first proposed the free radical reaction hypothesis in 2020 (73), which was later completed by Hu et al. in 2023 (74).

Beyond oxygen, free radicals can also react with iron-containing proteins and other biomolecules, generating unstable active iron species and organic peroxyl radicals, causing great damage on irradiated cellsa (75, 76). Under FLASH-RT conditions, the spatial clustering of radicals offered another way to mitigate these damages. Due to the high concentration, free radicals meet each other easily and start self-recombination reaction, leaving fewer radicals to cause damage (73, 77, 78). However, since tumor tissues have accumulated more antioxidants in cells, most free radicals are combining with the antioxidants, making the protective effect of FLASH-RT less distinctive (74) (**Figure 1**).

Current evidences confirm the reduced free radical output after FLASH-RT (79) and the decreased lipid peroxidation levels (80, 81), supporting the free radical mechanism. Nevertheless, further studies are still needed to validate this hypothesis with additional experimental evidence.

Similar to FLASH-induced transient hypoxia, the mechanism of the free radical alteration is also triggered by the characteristic ultra-high dose delivery within an extremely brief timeframe in FLASH-RT. That means FLASH-RT generates an ultra-high concentration of reducing radicals within a second, creating a distinct temporal distribution pattern of radicals compared to CONV-RT, which produces lower radical concentrations slowly over an extended duration. The oxygen depletion theory is invoked when the high concentration of radicals generated by FLASH reacts with oxygen, rapidly inducing oxygen exhaustion. Conversely, the radical recombination hypothesis applies when these concentrated radicals collide and undergo self-recombination reactions.

### 3.3 Mitochondrial hypothesis

As one of the key organelles, mitochondria play an important role in cell apoptosis after radiation. The mitochondrial hypothesis is fundamentally interconnected with FLASH-induced oxygen depletion. The large amount of reductive free radicals produced by FLASH-RT react with almost all the oxygen in the tissue and generated a large amount of ROS. This leads to a sharp increase in the permeability of the mitochondrial membrane (82), with a large amount of reactive oxygen species appearing inside the mitochondria, making the release of cytochrome and the activation of the apoptotic pathway possible (63). In fact, the mitochondrial response can be regarded as a unique consequence of the rapid oxygen depletion of FLASH-RT.

In 2021, Han et al. first discovered the link between mitochondrial activity and the emergence of the FLASH effect (83). Subsequently, further research by Guo et al. verified the protective effect of FLASH-RT on the morphology and structure of mitochondrial in normal tissues, and proposed the mitochondrial hypothesis (84).This hypothesis was later revised and refined by Jianfeng L et al (85).

According to the mitochondrial hypothesis, mitochondria produce excessive ROS after FLASH-RT, which destroys the electron transport chain (ETC) and induces the cytochrome c detachment (85). The detached cytochrome c enters the cytoplasm and triggering an apoptotic response while inhibiting the inflammatory responses, thereby protecting normal tissues from radiation damage (63). However, due to the abnormal of electron transport chains in tumor tissue, cytochrome c in tumor cells is unable to detach normally after FLASH-RT. Consequently, FLASH-RT fails to suppress the inflammatory responses and results in no sparing effect (85). In that case, apoptosis occurs in most of the normal cells while necrosis is more common among tumor cells (84) (**Figure 1**).

Although a relatively complete theory has been proposed, experimental evidences of this hypothesis is still insufficient. In the future, more experiments are needed to refine and validate the mitochondrial hypothesis. Furthermore, recent studies have revealed that FLASH-RT can activate mitochondrial-associated glycolytic pathways while suppressing oxidative phosphorylation, thereby enhancing tumor radiosensitivity under hypoxic conditions (86). However, the implications of FLASH-induced mitochondrial metabolic reprogramming for normal tissue protection remain unclear and urgently require further experimental investigation.

### 3.4 DNA integrity hypothesis

After FLASH-RT occurs, DNA will simultaneously suffer direct damage caused by direct impact from high-energy particles and indirect damage mediated by free radical attacks. Due to the high dose rate characteristic of FLASH-RT, the spatiotemporal distribution of high-energy particles and free radicals generated during radiotherapy has changed, showing a concentrated appearance within a short period of time. Just as the oxygen depletion theory and the free radical reaction hypothesis suggest, the highly concentrated appearance of free radicals will instead exhibit fewer destruction.

Multiple studies have shown that FLASH-RT produce fewer DNA damage compared with CONV-RT. That include reduced single-strand breaks (SSBs) (87), gathered double-strand breaks (DSBs), less dicentric chromosomes (88) and micronuclei (67). It is worth noting that the study of Daisuke et al. suggest although the number of SSBs under FLASH-RT decreased, the quantity of DSBs never changed (88), only appeared more concentrated (67)(**Figure 1**). However, Alan et al. indicating that the number of DSBs after FLASH indeed decreased (89). Despite the different results, both experiments used proton beams as irradiation ray, plasmids as experimental subjects, even the irradiation doses and dose rates were highly similar. Collectively, the difference of these results may be attributed to the variations in testing time, during which DNA reparation had already start, thereby affecting the detection outcomes. In subsequent experiments, more precise control of sampling time and electrophoresis separation should be utilized to ensure the detection of all DSBs. Anyhow, both the reduced quantity and the concentrated presence of DSBs is conducive to the DNA repairment (90), which helps to the radioprotective effect and lead to a less pronounced G2/M-accumulation (91).

After DNA damage induction, irradiated cells will rapidly activate the non-homologous end joining (NHEJ) pathway to ligate broken DNA ends at DSBs, while SSBs will be repaired by poly-ADP-ribose polymerase pathway. Under physiological conditions, most of DSB repairs are mediated by NHEJ, although this pathway is more error-prone (92). When entering the G2/M phase, irradiated cells will assemble the repair-associated complexes to DSB foci and execute end reception, then activate the homologous recombination repair (HDR) (93). The shorter G2/M arrest following FLASH-RT implies that more concentrated DSB clusters reduce the time required for repair complex recruitment to DSB foci, thereby enhancing repair efficiency (93). Currently, the choice between the HDR and NHEJ pathways in cells mainly depends on whether end resection occurs and whether sister chromatids exist (94). However, the enhancement of HDR efficiency by clustered DSBs may enable more DSBs to undergo high-fidelity repair, thereby reducing radiation-induced damage. While in tumor tissues, metabolic dysregulation compromises the HDR pathway (95), making the highly concentrated DSB cannot leverage HDR-mediated precision repair to mitigate the misincorporation risks, rendering this protective mechanism ineffective in malignancies.

However, the above conjectures still require further experimental validation. Given that current research on FLASH-induced DNA damage primarily focuses on quantification of DNA damage foci, more comprehensive studies addressing DNA repair pathways and repair therapeutics are urgently needed.

### 3.5 Other hypothesis

In addition to the above four theories, there are more hypotheses that are not yet perfect, but are still of equally importance.

Accumulating experimental evidence demonstrates that FLASH-RT not only attenuates inflammatory response in normal tissues (44, 47), but also activates the immune cells in tumor tissues (96, 97). As predicted by the mitochondrial hypothesis, the suppressed inflammatory response in normal tissues may result from the higher rate of apoptosis mediated by cytochrome c leakage (63) (**Figure 1**). In tumor tissues, the enhanced inflammatory response appears to be mediated through CD8+ T lymphocytes recruitment and TGF-β secretion upregulation, although the underlying mechanisms remain unclear. In addition, the radioprotection and stimulation of immune system is also an important reason for the enhanced tumor killing of FLASH-RT (98, 100). Multiple experiments have confirmed improved survival of both tissue-resident (99) and circulating immune cells following FLASH-RT (59, 100). When combining tissue-sparing effects with enhanced tumor immunogenicity, FLASH-RT is likely to have a promising future when combining with immunotherapy (100). By combining ICI-mediated blockade of immune checkpoints (e.g., PD-1) with FLASH-enhanced immune cell infiltration in tumor tissues, the immune system’s ability to eliminate tumor cells is further potentiated. Meanwhile, the mitigated normal tissue inflammation after FLASH-RT also reduces the risk of off-target toxicity associated with immune checkpoints inhibition. However, further experiments are still required to better confirm the feasibility and security of this combination (101).

In addition to immune cells, FLASH-RT also preserves the surrounding vascular and mucosal structures, thereby maintaining the tissue architecture and nutrient supply in irradiated organs (9). And that many contribute to the post-radiation repairment. Studies demonstrate that FLASH-RT can significantly minimizes the stem cell damage (26) and attenuates radiation-induced cellular senescence (26), thereby reducing the late toxicity leaded by stem cell depletion. Moreover, FLASH-RT-mediated activation of microstructural components including myosin light chains contributes significantly to tumor microenvironment modulation and DNA damage mitigation (42). Beyond the conventional mitochondrial hypothesis, emerging research have also pointed out that the specific protection of FLASH on the structure and function of mitochondria in normal tissues is likely to be another key mechanism for reducing the toxicity of normal tissues in FLASH-RT (102). However, the rigorousness and systematicity of these hypotheses remain insufficient, requiring more experiments for further validation.

Elucidating the mechanisms underlying the FLASH effect serves not only to clarify the complex interrelationships among influencing factors, but also to establish the theoretical foundation for FLASH-RT’s clinical applicability. Among current hypotheses, only the oxygen depletion, free radicals, DNA integrity, and mitochondrial hypotheses provide relatively comprehensive molecular-level explanations of the FLASH effect. The remaining hypotheses are mostly confined to the cellular level, offering supplementary evidence rather than fundamental mechanistic insights. In subsequent studies, further research is needed to investigate these cellular-level hypotheses and delve into their molecular mechanisms.

## 4 Conclusions and prospects

As an innovative radiotherapy modality, FLASH-RT shows distinct advantages including normal tissue sparing, treatment time reduction and OER reduction in tumor microenvironment. To facilitate research progress and clinical translation of FLASH-RT, it is essential to systematically investigate both the influencing factors and the underlying mechanisms of the FLASH effect. These studies will help to clarify the optimal dose rates and treatment parameters of FLASH-RT, which are crucial for future clinical adoption and implementation.

Recent studies have identified multiple factors influencing the FLASH effect, but inconsistent experimental results suggest numerous potential determinants remain to be discovered. A variety of mechanistic hypotheses have been proposed, such as oxygen depletion, free radical reaction, DNA integrity and mitochondrial hypothesis. However, none of these mechanisms can comprehensively explain all FLASH-related phenomena, indicating that these mechanisms are still uncompleted and requiring further validation and refinement. With further exploration of the influencing factors and underlying mechanisms of FLASH effect, the broad clinical adoption of FLASH-RT is poised to become an inevitable trend in radiation oncology.

## References

[1] Bray F Laversanne M Sung H Ferlay J Siegel RL Soerjomataram I . Global cancer statistics 2022: GLOBOCAN estimates of incidence and mortality worldwide for 36 cancers in 185 countries. CA Cancer J Clin. (2024) 74:229–63. doi: 10.3322/caac.21834, PMID: 38572751

[2] Siegel RL Giaquinto AN Jemal A . Cancer statistics, 2024. CA Cancer J Clin. (2024) 74:12–49. doi: 10.3322/caac.21820, PMID: 38230766

[3] Herrera FG Bourhis J Coukos G . Radiotherapy combination opportunities leveraging immunity for the next oncology practice. CA Cancer J Clin. (2017) 67:65–85. doi: 10.3322/caac.21358, PMID: 27570942

[4] Moding EJ Kastan MB Kirsch DG . Strategies for optimizing the response of cancer and normal tissues to radiation. Nat Rev Drug Discov. (2013) 12:526–42. doi: 10.1038/nrd4003, PMID: 23812271 PMC3906736

[5] Stemkens B Paulson ES Tijssen RHN . Nuts and bolts of 4D-MRI for radiotherapy. Phys Med Biol. (2018) 63:21TR01. doi: 10.1088/1361-6560/aae56d, PMID: 30272573

[6] Prabhu RS Dhakal R Piantino M Bahar N Meaders KS Fasola CE . Volumetric modulated arc therapy (VMAT) craniospinal irradiation (CSI) for children and adults: A practical guide for implementation. Pract Radiat Oncol. (2022) 12:e101–9:2. doi: 10.1016/j.prro.2021.11.005, PMID: 34848379

[7] Coon AB Dickler A Kirk MC Liao Y Shah AP Strauss JB . Tomotherapy and multifield intensity-modulated radiotherapy planning reduce cardiac doses in left-sided breast cancer patients with unfavorable cardiac anatomy. Int J Radiat oncology biology Phys. (2010) 78:104–10. doi: 10.1016/j.ijrobp.2009.07.1705, PMID: 20004529

[8] Caines R Sisson NK Rowbottom CG . 4DCT and VMAT for lung patients with irregular breathing. J Appl Clin Med Phys. (2022) 23:e13453. doi: 10.1002/acm2.13453, PMID: 34816564 PMC8803302

[9] Favaudon V Caplier L Monceau V Pouzoulet F Sayarath M Fouillade C . Ultrahigh dose-rate FLASH irradiation increases the differential response between normal and tumor tissue in mice. Sci Transl Med. (2014) 6:245ra93. doi: 10.1126/scitranslmed.3008973, PMID: 25031268

[10] Dewey DL Boag JW . Modification of the oxygen effect when bacteria are given large pulses of radiation. Nature. (1959) 183:1450–1. doi: 10.1038/1831450a0, PMID: 13657161

[11] Town CD . Effect of high dose-rates on survival of mammalian cells. Nature. (1967) 215:847–8. doi: 10.1038/215847a0, PMID: 6049731

[12] Beyreuther E Brand M Hans S Hideghéty K Karsch L Leßmann E . Feasibility of proton FLASH effect tested by zebrafish embryo irradiation. Radiotherapy Oncol. (2019) 139:46–50. doi: 10.1016/j.radonc.2019.06.024, PMID: 31266652

[13] Konradsson E Arendt ML Bastholm Jensen K Børresen B Hansen AE Bäck S . Establishment and initial experience of clinical FLASH radiotherapy in canine cancer patients. Front Oncol. (2021) 11:658004. doi: 10.3389/fonc.2021.658004, PMID: 34055624 PMC8155542

[14] Vozenin M-C De Fornel P Petersson K Favaudon V Jaccard M Germond JF . The advantage of FLASH radiotherapy confirmed in mini-pig and cat-cancer patients. Clin Cancer Res. (2019) 25:35–42. doi: 10.1158/1078-0432.CCR-17-3375, PMID: 29875213

[15] Bourhis J Sozzi WJ Jorge PG Gaide O Bailat C Duclos F . Treatment of a first patient with FLASH-radiotherapy. Radiotherapy Oncol. (2019) 139:18–22. doi: 10.1016/j.radonc.2019.06.019, PMID: 31303340

[16] Gaide O Herrera F Jeanneret Sozzi W Gonçalves Jorge P Kinj R Bailat C . Comparison of ultra-high versus conventional dose rate radiotherapy in a patient with cutaneous lymphoma. Radiotherapy Oncol. (2022) 174:87–91. doi: 10.1016/j.radonc.2021.12.045, PMID: 34998899

[17] Mascia AE Daugherty EC Zhang Y Lee E Xiao Z Sertorio M . Proton FLASH radiotherapy for the treatment of symptomatic bone metastases: the FAST-01 nonrandomized trial. JAMA Oncol. (2023) 9:62–9. doi: 10.1001/jamaoncol.2022.5843, PMID: 36273324 PMC9589460

[18] Daugherty EC Zhang Y Xiao Z Mascia AE Sertorio M Woo J . FLASH radiotherapy for the treatment of symptomatic bone metastases in the thorax (FAST-02): protocol for a prospective study of a novel radiotherapy approach. Radiat Oncol (London England). (2024) 19:34. doi: 10.1186/s13014-024-02419-4, PMID: 38475815 PMC10935811

[19] Kinj Rémy Gaide O Jeanneret-Sozzi W Dafni U Viguet-Carrin S Sagittario E . Randomized phase II selection trial of FLASH and conventional radiotherapy for patients with localized cutaneous squamous cell carcinoma or basal cell carcinoma: A study protocol. Clin Trans Radiat Oncol. (2024) 45:100743. doi: 10.1016/j.ctro.2024.100743, PMID: 38362466 PMC10867306

[20] Thariat J Hannoun-Levi JM Sun Myint A Vuong T Gérard JP . Past, present, and future of radiotherapy for the benefit of patients. Nature reviews. Clin Oncol. (2013) 10:52–60. doi: 10.1038/nrclinonc.2012.203, PMID: 23183635

[21] Diffenderfer ES Verginadis II Kim MM Shoniyozov K Velalopoulou A Goia D . Design, implementation, and *in vivo* validation of a novel proton FLASH radiation therapy system. Int J Radiat oncology biology Phys. (2020) 106:440–8. doi: 10.1016/j.ijrobp.2019.10.049, PMID: 31928642 PMC7325740

[22] Montay-Gruel P Bouchet A Jaccard M Patin D Serduc R Aim W . X-rays can trigger the FLASH effect: Ultra-high dose-rate synchrotron light source prevents normal brain injury after whole brain irradiation in mice. Radiotherapy Oncol. (2018) 129:582–8. doi: 10.1016/j.radonc.2018.08.016, PMID: 30177374

[23] Gao F Yang Y Zhu H Wang J Xiao D Zhou Z . First demonstration of the FLASH effect with ultrahigh dose rate high-energy X-rays. Radiotherapy Oncol. (2022) 166:44–50. doi: 10.1016/j.radonc.2021.11.004, PMID: 34774651

[24] Tinganelli W Puspitasari-Kokko A Sokol O Helm A Simoniello P Schuy C . FLASH bragg-peak irradiation with a therapeutic carbon ion beam: first *in vivo* results. Int J Radiat oncology biology Phys. (2024) 121:1282–92. doi: 10.1016/j.ijrobp.2024.11.089, PMID: 39608612

[25] Zhang Q Gerweck LE Cascio E Gu L Yang Q Dong X . Absence of tissue-sparing effects in partial proton FLASH irradiation in murine intestine. Cancers. (2023) 15:2269. doi: 10.3390/cancers15082269, PMID: 37190197 PMC10137009

[26] Fouillade C Curras-Alonso S Giuranno L Quelennec E Heinrich S Bonnet-Boissinot S . FLASH irradiation spares lung progenitor cells and limits the incidence of radio-induced senescence. Clin Cancer Res. (2020) 26:1497–506. doi: 10.1158/1078-0432.CCR-19-1440, PMID: 31796518

[27] Sunnerberg JP Tavakkoli AD Petusseau AF Daniel NJ Sloop AM Schreiber WA . Oxygen consumption *in vivo* by ultra-high dose rate electron irradiation depends upon baseline tissue oxygenation. Int J Radiat oncology biology Phys. (2024) 121:1053–62. doi: 10.1016/j.ijrobp.2024.10.018, PMID: 39461597 PMC11850185

[28] Beckers C Pruschy M Vetrugno I . Tumor hypoxia and radiotherapy: A major driver of resistance even for novel radiotherapy modalities. Semin Cancer Biol. (2024) 98:19–30. doi: 10.1016/j.semcancer.2023.11.006, PMID: 38040401

[29] Chaudhary P Gwynne DC Odlozilik B McMurray A Milluzzo G Maiorino C . Development of a portable hypoxia chamber for ultra-high dose rate laser-driven proton radiobiology applications. Radiat Oncol (London England). (2022) 17:77. doi: 10.1186/s13014-022-02024-3, PMID: 35428301 PMC9013042

[30] Tan HS Teo KBK Dong L Friberg A Koumenis C Diffenderfer E . Modeling ultra-high dose rate electron and proton FLASH effect with the physicochemical approach. Phys Med Biol. (2023) 68:14. doi: 10.1088/1361-6560/ace14d, PMID: 37352867 PMC10472835

[31] Levy K Natarajan S Wang J Chow S Eggold JT Loo PE . Abdominal FLASH irradiation reduces radiation-induced gastrointestinal toxicity for the treatment of ovarian cancer in mice. Sci Rep. (2020) 10:21600. doi: 10.1038/s41598-020-78017-7, PMID: 33303827 PMC7728763

[32] Zhu H Xie D Yang Y Huang S Gao X Peng Y . Radioprotective effect of X-ray abdominal FLASH irradiation: Adaptation to oxidative damage and inflammatory response may be benefiting factors. Med Phys. (2022) 49:4812–22. doi: 10.1002/mp.15680, PMID: 35451077

[33] Valdés Zayas A Kumari N Liu K Neill D Delahoussaye A Gonçalves Jorge P . Independent reproduction of the FLASH effect on the gastrointestinal tract: A multi-institutional comparative study. Cancers. (2023) 15:2121. doi: 10.3390/cancers15072121, PMID: 37046782 PMC10093322

[34] Ruan J-L Lee C Wouters S Tullis IDC Verslegers M Mysara M . Irradiation at ultra-high (FLASH) dose rates reduces acute normal tissue toxicity in the mouse gastrointestinal system. Int J Radiat oncology biology Phys. (2021) 111:1250–61. doi: 10.1016/j.ijrobp.2021.08.004, PMID: 34400268 PMC7612009

[35] Lim TL Morral C Verginadis II Kim K Luo L Foley CJ . Early inflammation and interferon signaling direct enhanced intestinal crypt regeneration after proton FLASH radiotherapy. bioRxiv: preprint server Biol. (2024), 2024.08.16.608284. doi: 10.1101/2024.08.16.608284, PMID: 39229237 PMC11370362

[36] Velalopoulou A Karagounis IV Cramer GM Kim MM Skoufos G Goia D . FLASH proton radiotherapy spares normal epithelial and mesenchymal tissues while preserving sarcoma response. Cancer Res. (2021) 81:4808–21. doi: 10.1158/0008-5472.CAN-21-1500, PMID: 34321243 PMC8715480

[37] Singers Sørensen B Krzysztof Sitarz M Ankjærgaard C Johansen J Andersen CE Kanouta E . *In vivo* validation and tissue sparing factor for acute damage of pencil beam scanning proton FLASH. Radiotherapy Oncol. (2022) 167:109–15. doi: 10.1016/j.radonc.2021.12.022, PMID: 34953933

[38] Soto LA Casey KM Wang J Blaney A Manjappa R Breitkreutz D . FLASH irradiation results in reduced severe skin toxicity compared to conventional-dose-rate irradiation. Radiat Res. (2020) 194:618–24. doi: 10.1667/RADE-20-00090, PMID: 32853385 PMC7855987

[39] Zhang Q Gerweck LE Cascio E Yang Q Huang P Niemierko A . Proton FLASH effects on mouse skin at different oxygen tensions. Phys Med Biol. (2023) 68:5. doi: 10.1088/1361-6560/acb888, PMID: 36731139 PMC11164666

[40] Poulsen PR Johansen JG Sitarz MK Kanouta E Kristensen L Grau C . Oxygen enhancement ratio-weighted dose quantitatively describes acute skin toxicity variations in mice after pencil beam scanning proton FLASH irradiation with changing doses and time structures. Int J Radiat oncology biology Phys. (2024) 120:276–86. doi: 10.1016/j.ijrobp.2024.02.050, PMID: 38462015

[41] Adrian G Konradsson E Beyer S Wittrup A Butterworth KT McMahon SJ . Cancer cells can exhibit a sparing FLASH effect at low doses under normoxic *in vitro*-conditions. Front Oncol. (2021) 11:686142. doi: 10.3389/fonc.2021.686142, PMID: 34395253 PMC8358772

[42] Kim Y-E Gwak SH Hong BJ Oh JM Choi HS Kim MS . Effects of ultra-high doserate FLASH irradiation on the tumor microenvironment in lewis lung carcinoma: role of myosin light chain. Int J Radiat oncology biology Phys. (2021) 109:1440–53. doi: 10.1016/j.ijrobp.2020.11.012, PMID: 33186615

[43] Montay-Gruel P Petersson K Jaccard M Boivin G Germond JF Petit B . Irradiation in a FLASH: Unique sparing of memory in mice after whole brain irradiation with dose rates above 100Gy/s. Radiotherapy Oncol. (2017) 124:365–9. doi: 10.1016/j.radonc.2017.05.003, PMID: 28545957

[44] Simmons DA Lartey FM Schüler E Rafat M King G Kim A . Reduced cognitive deficits after FLASH irradiation of whole mouse brain are associated with less hippocampal dendritic spine loss and neuroinflammation. Radiotherapy Oncol. (2019) 139:4–10. doi: 10.1016/j.radonc.2019.06.006, PMID: 31253467

[45] Montay-Gruel P Acharya MM Petersson K Alikhani L Yakkala C Allen BD . Long-term neurocognitive benefits of FLASH radiotherapy driven by reduced reactive oxygen species. Proc Natl Acad Sci United States America. (2019) 116:10943–51. doi: 10.1073/pnas.1901777116, PMID: 31097580 PMC6561167

[46] Dokic I Meister S Bojcevski J Tessonnier T Walsh D Knoll M . Neuroprotective effects of ultra-high dose rate FLASH bragg peak proton irradiation. Int J Radiat Oncol Biol Phys. (2022) 113:614–23. doi: 10.1016/j.ijrobp.2022.02.020, PMID: 35196536 PMC11034835

[47] Dokic I Moustafa M Tessonnier T Meister S Ciamarone F Akbarpour M . Ultra-high dose rate helium ion beams: minimizing brain tissue damage while preserving tumor control. Mol Cancer Ther. (2024) 24:763–71. doi: 10.1158/1535-7163.MCT-24-0536, PMID: 39739545 PMC12046314

[48] Jansen J Kimbler A Drayson O Lanz B Mosso J Grilj V . Ex vivo brain MRI to assess conventional and FLASH brain irradiation effects. Radiotherapy Oncol. (2025) 208:110894. doi: 10.1016/j.radonc.2025.110894, PMID: 40233872 PMC12919612

[49] Drayson OGG Melemenidis S Katila N Viswanathan V Kramár EA Zhang R . A multi-institutional study to investigate the sparing effect after whole brain electron FLASH in mice: Reproducibility and temporal evolution of functional, electrophysiological, and neurogenic endpoints. Radiotherapy Oncol. (2024) 201:110534. doi: 10.1016/j.radonc.2024.110534, PMID: 39293721 PMC11588524

[50] Karsch L Pawelke J Brand M Hans S Hideghéty K Jansen J . Beam pulse structure and dose rate as determinants for the FLASH effect observed in zebrafish embryo. Radiotherapy Oncol. (2022) 173:49–54. doi: 10.1016/j.radonc.2022.05.025, PMID: 35661675

[51] Böhlen TT Germond JF Bourhis J Vozenin MC Ozsahin EM Bochud F . Normal tissue sparing by FLASH as a function of single-fraction dose: A quantitative analysis. Int J Radiat oncology biology Phys. (2022) 114:1032–44. doi: 10.1016/j.ijrobp.2022.05.038, PMID: 35810988

[52] Horst F Germond JF Bourhis J Vozenin MC Ozsahin EM Bochud F . Dose and dose rate dependence of the tissue sparing effect at ultra-high dose rate studied for proton and electron beams using the zebrafish embryo model. Radiotherapy Oncol. (2024) 194:110197. doi: 10.1016/j.radonc.2024.110197, PMID: 38447870

[53] Limoli CL Kramár EA Almeida A Petit B Grilj V Baulch JE . The sparing effect of FLASH-RT on synaptic plasticity is maintained in mice with standard fractionation. Radiotherapy Oncol. (2023) 186:109767. doi: 10.1016/j.radonc.2023.109767, PMID: 37385377 PMC11045040

[54] Dai Y Liang R Wang J Zhang J Wu D Zhao R . Fractionated FLASH radiation in xenografted lung tumors induced FLASH effect at a split dose of 2 Gy. Int J Radiat Biol. (2023) 99:1542–9. doi: 10.1080/09553002.2023.2194403, PMID: 36952604

[55] Sørensen BS Kanouta E Ankjærgaard C Kristensen L Johansen JG Sitarz MK . Proton FLASH: impact of dose rate and split dose on acute skin toxicity in a murine model. Int J Radiat oncology biology Phys. (2024) 120:265–75. doi: 10.1016/j.ijrobp.2024.04.071, PMID: 38750904

[56] Liu K Waldrop T Aguilar E Mims N Neill D Delahoussaye A . Redefining FLASH radiation therapy: the impact of mean dose rate and dose per pulse in the gastrointestinal tract. Int J Radiat oncology biology Phys. (2025) 121:1063–76. doi: 10.1016/j.ijrobp.2024.10.009, PMID: 39424078

[57] Rohrer Bley C Wolf F Gonçalves Jorge P Grilj V Petridis I Petit B . Dose- and volume-limiting late toxicity of FLASH radiotherapy in cats with squamous cell carcinoma of the nasal planum and in mini pigs. Clin Cancer Res. (2022) 28:3814–23. doi: 10.1158/1078-0432.CCR-22-0262, PMID: 35421221 PMC9433962

[58] Liu K Titt U Esplen N Connell L Konradsson E Yang M . Discordance in acute gastrointestinal toxicity between synchrotron-based proton and linac-based electron ultra-high dose rate irradiation. Int J Radiat Oncol Biol Phys. (2024) 122:491–501. doi: 10.1101/2024.09.04.611307, PMID: 39862897 PMC13383351

[59] Jin JY Gu A Wang W Oleinick NL Machtay M Spring Kong FM . Ultra-high dose rate effect on circulating immune cells: A potential mechanism for FLASH effect? Radiotherapy oncology: J Eur Soc Ther Radiol Oncol. (2020) 149:55–62. doi: 10.1016/j.radonc.2020.04.054, PMID: 32387486 PMC7442672

[60] Iturri L Bertho A Lamirault C Juchaux M Gilbert C Espenon J . Proton FLASH radiation therapy and immune infiltration: evaluation in an orthotopic glioma rat model. Int J Radiat oncology biology Phys. (2023) 116:655–65. doi: 10.1016/j.ijrobp.2022.12.018, PMID: 36563907

[61] Thomas W Sunnerberg J Reed M Gladstone DJ Zhang R Harms J . Proton and electron ultrahigh-dose-rate isodose irradiations produce differences in reactive oxygen species yields. Int J Radiat oncology biology Phys. (2024) 118:262–7. doi: 10.1016/j.ijrobp.2023.07.042, PMID: 37558097 PMC10843497

[62] Kacem H Psoroulas S Boivin G Folkerts M Grilj V Lomax T . Comparing radiolytic production of H2O2 and development of Zebrafish embryos after ultra high dose rate exposure with electron and transmission proton beams. Radiotherapy Oncol. (2022) 175:197–202. doi: 10.1016/j.radonc.2022.07.011, PMID: 35868604

[63] Shi X Yang Y Zhang W Wang J Xiao D Ren H . FLASH X-ray spares intestinal crypts from pyroptosis initiated by cGAS-STING activation upon radioimmunotherapy. Proc Natl Acad Sci United States America. (2022) 119:e2208506119. doi: 10.1073/pnas.2208506119, PMID: 36256824 PMC9618056

[64] Tinganelli W Weber U Puspitasari A Simoniello P Abdollahi A Oppermann J . FLASH with carbon ions: Tumor control, normal tissue sparing, and distal metastasis in a mouse osteosarcoma model. Radiotherapy Oncol. (2022) 175:185–90. doi: 10.1016/j.radonc.2022.05.003, PMID: 35537606

[65] Tashiro M Yoshida Y Oike T Nakao M Yusa K Hirota Y . First human cell experiments with FLASH carbon ions. Anticancer Res. (2022) 42:2469–77. doi: 10.21873/anticanres.15725, PMID: 35489744

[66] Lin B Gao F Yang Y Wu D Zhang Y Feng G . FLASH radiotherapy: history and future. Front Oncol. (2021) 11:644400. doi: 10.3389/fonc.2021.644400, PMID: 34113566 PMC8185194

[67] Ma Y Zhang W Zhao Z Lv J Chen J Yan X . Current views on mechanisms of the FLASH effect in cancer radiotherapy. Natl Sci Rev. (2024) 11:nwae350. doi: 10.1093/nsr/nwae350, PMID: 39479528 PMC11523052

[68] Barendsen GW Koot CJ Van Kersen GR Bewley DK Field SB Parnell CJ . The effect of oxygen on impairment of the proliferative capacity of human cells in culture by ionizing radiations of different LET. Int J Radiat Biol related Stud physics chemistry Med. (1966) 10:317–27. doi: 10.1080/09553006614550421, PMID: 5297012

[69] Cao Xu Zhang R Esipova TV Allu SR Ashraf R Rahman M . Quantification of oxygen depletion during FLASH irradiation *in vitro* and *in vivo*. Int J Radiat oncology biology Phys. (2021) 111:240–8. doi: 10.1016/j.ijrobp.2021.03.056, PMID: 33845146 PMC8338745

[70] Scarmelotto A Delprat V Michiels C Lucas S Heuskin AC . The oxygen puzzle in FLASH radiotherapy: A comprehensive review and experimental outlook. Clin Trans Radiat Oncol. (2024) 49:100860. doi: 10.1016/j.ctro.2024.100860, PMID: 39381632 PMC11458961

[71] Hornsey S Bewley DK . Hypoxia in mouse intestine induced by electron irradiation at high dose-rates. Int J Radiat Biol related Stud physics chemistry Med. (1971) 19:479–83. doi: 10.1080/09553007114550611, PMID: 5314348

[72] Tinganelli W Sokol O Quartieri M Puspitasari A Dokic I Abdollahi A . Ultra-high dose rate (FLASH) carbon ion irradiation: dosimetry and first cell experiments. Int J Radiat oncology biology Phys. (2022) 112:4. doi: 10.1016/j.ijrobp.2021.11.020, PMID: 34813912

[73] Labarbe R Hotoiu L Barbier J Favaudon V . A physicochemical model of reaction kinetics supports peroxyl radical recombination as the main determinant of the FLASH effect. Radiotherapy Oncol. (2020) 153:303–10. doi: 10.1016/j.radonc.2020.06.001, PMID: 32534957

[74] Hu A Qiu R Li WB Zhou W Wu Z Zhang H . Radical recombination and antioxidants: a hypothesis on the FLASH effect mechanism. Int J Radiat Biol. (2023) 99:620–8. doi: 10.1080/09553002.2022.2110307, PMID: 35938944

[75] Qian SY Buettner GR . Iron and dioxygen chemistry is an important route to initiation of biological free radical oxidations: an electron paramagnetic resonance spin trapping study. Free Radical Biol Med. (1999) 26:1447–56. doi: 10.1016/s0891-5849(99)00002-7, PMID: 10401608

[76] Tudek B Zdżalik-Bielecka D Tudek A Kosicki K Fabisiewicz A Speina E . Lipid peroxidation in face of DNA damage, DNA repair and other cellular processes. Free Radical Biol Med. (2017) 107:77–89. doi: 10.1016/j.freeradbiomed.2016.11.043, PMID: 27908783

[77] Wardman P . Radiotherapy using high-intensity pulsed radiation beams (FLASH): A radiation-chemical perspective. Radiat Res. (2020) 194:607–17. doi: 10.1667/RADE-19-00016, PMID: 33348369

[78] Derksen L Flatten V Engenhart-Cabillic R Zink K Baumann KS . A method to implement inter-track interactions in Monte Carlo simulations with TOPAS-nBio and their influence on simulated radical yields following water radiolysis. Phys Med Biol. (2023) 68:13. doi: 10.1088/1361-6560/acdc7d, PMID: 37285861

[79] Espinosa-Rodriguez A Sanchez-Parcerisa D Ibáñez P Vera-Sánchez JA Mazal A Fraile LM . Radical production with pulsed beams: understanding the transition to FLASH. Int J Mol Sci. (2022) 23:13484. doi: 10.3390/ijms232113484, PMID: 36362271 PMC9656621

[80] Portier L Daira P Fourmaux B Heinrich S Becerra M Fouillade C . Differential remodeling of the oxylipin pool after FLASH versus conventional dose-rate irradiation *in vitro* and *in vivo*. Int J Radiat oncology biology Phys. (2024) 119:1481–92. doi: 10.1016/j.ijrobp.2024.01.210, PMID: 38340776

[81] Froidevaux P Grilj V Bailat C Walter Reiner Geyer François Bochud Marie-Catherine Vozenin . FLASH irradiation does not induce lipid peroxidation in lipids micelles and liposomes. Radiat Phys Chem. (2023) 205:110733. doi: 10.1016/j.radphyschem.2022.110733

[82] Vringer E Stephen WGT . Mitochondria and inflammation: cell death heats up. Front Cell Dev Biol. (2019) 7:100. doi: 10.3389/fcell.2019.00100, PMID: 31316979 PMC6610339

[83] Han J Mei Z Lu C Qian J Liang Y Sun X . Ultra-high dose rate FLASH irradiation induced radio-resistance of normal fibroblast cells can be enhanced by hypoxia and mitochondrial dysfunction resulting from loss of cytochrome C. Front Cell Dev Biol. (2021) 9:672929. doi: 10.3389/fcell.2021.672929, PMID: 33996831 PMC8121317

[84] Guo Z Buonanno M Harken A Zhou G Hei TK . Mitochondrial damage response and fate of normal cells exposed to FLASH irradiation with protons. Radiat Res. (2022) 197:569–82. doi: 10.1667/RADE-21-00181.1, PMID: 35290449 PMC9241019

[85] Jianfeng L Jianhan S Yunbin L Juntao L Di W Yiyu F . FLASH irradiation regulates IFN-β induction by mtDNA via cytochrome c leakage. bioRxiv. (2024) 04:10. doi: 10.1101/2024.04.10.588811

[86] Leavitt RJ Almeida A Grilj V Montay-Gruel P Godfroid C Petit B . Acute hypoxia does not alter tumor sensitivity to FLASH radiation therapy. Int J Radiat oncology biology Phys. (2024) 119:1493–505. doi: 10.1016/j.ijrobp.2024.02.015, PMID: 38387809

[87] Ohsawa D Hiroyama Y Kobayashi A Kusumoto T Kitamura H Hojo S . DNA strand break induction of aqueous plasmid DNA exposed to 30 MeV protons at ultra-high dose rate. J Radiat Res. (2022) 63:255–60. doi: 10.1093/jrr/rrab114, PMID: 34952540 PMC8944314

[88] Garty G Obaid R Deoli N Royba E Tan Y Harken AD . Ultra-high dose rate FLASH irradiator at the radiological research accelerator facility. Sci Rep. (2022) 12:22149. doi: 10.1038/s41598-022-19211-7, PMID: 36550150 PMC9780319

[89] Perstin A Poirier Y Sawant A Tambasco M . Quantifying the DNA-damaging effects of FLASH irradiation with plasmid DNA. Int J Radiat oncology biology Phys. (2022) 113:437–47. doi: 10.1016/j.ijrobp.2022.01.049, PMID: 35124135

[90] Schrank BR Aparicio T Li Y Chang W Chait BT Gundersen GG . Nuclear ARP2/3 drives DNA break clustering for homology-directed repair. Nature. (2018) 559:61–6. doi: 10.1038/s41586-018-0237-5, PMID: 29925947 PMC6145447

[91] Auer S Hable V Greubel C Drexler GA Schmid TE Belka C . Survival of tumor cells after proton irradiation with ultra-high dose rates. Radiat Oncol (London England). (2011) 6:139. doi: 10.1186/1748-717X-6-139, PMID: 22008289 PMC3215966

[92] Symington LS Gautier J . Double-strand break end resection and repair pathway choice. Annu Rev Genet. (2011) 45:247–71. doi: 10.1146/annurev-genet-110410-132435, PMID: 21910633

[93] Jin Y-Y Zhang P Liu LL Zhao X Hu XQ Liu SZ . Enhancing homology-directed repair efficiency with HDR-boosting modular ssDNA donor. Nat Commun. (2024) 15:6843. doi: 10.1038/s41467-024-50788-x, PMID: 39122671 PMC11315919

[94] Symington LS . End resection at double-strand breaks: mechanism and regulation. Cold Spring Harbor Perspect Biol. (2014) 6:a016436. doi: 10.1101/cshperspect.a016436, PMID: 25085909 PMC4107989

[95] Chen L-L Xiong Y . Tumour metabolites hinder DNA repair. Nature. (2020) 582:492–4. doi: 10.1038/d41586-020-01569-1, PMID: 32572248 PMC7576997

[96] Ni H Reitman ZJ Zou W Akhtar MN Paul R Huang M . FLASH radiation reprograms lipid metabolism and macrophage immunity and sensitizes medulloblastoma to CAR-T cell therapy. Nat Cancer. (2025) 6:460–73. doi: 10.1038/s43018-025-00905-6, PMID: 39910249 PMC12244404

[97] Shukla S Saha T Rama N Acharya A Le T Bian F . Ultra-high dose-rate proton FLASH improves tumor control. Radiotherapy Oncol. (2023) 186:109741. doi: 10.1016/j.radonc.2023.109741, PMID: 37315577 PMC10527231

[98] Zhu H Xie D Wang Y Huang R Chen X Yang Y . Comparison of intratumor and local immune response between MV X-ray FLASH and conventional radiotherapies. Clin Trans Radiat Oncol. (2022) 38:138–46. doi: 10.1016/j.ctro.2022.11.005, PMID: 36425537 PMC9679438

[99] Chaklai A Canaday P O'Niel A Cucinotta FA Sloop A Gladstone D . Effects of UHDR and conventional irradiation on behavioral and cognitive performance and the percentage of ly6G+ CD45+ Cells in the hippocampus. Int J Mol Sci. (2023) 24:12497. doi: 10.3390/ijms241512497, PMID: 37569869 PMC10419899

[100] Galts A Hammi A . FLASH radiotherapy sparing effect on the circulating lymphocytes in pencil beam scanning proton therapy: impact of hypofractionation and dose rate. Phys Med Biol. (2024) 69:10.1088/1361-6560/ad144e. doi: 10.1088/1361-6560/ad144e, PMID: 38081067

[101] Wang Y Qi SN Bi N Li YX . FLASH radiotherapy combined with immunotherapy: From biological mechanisms to blockbuster therapeutics. Trans Oncol. (2025) 51:102183. doi: 10.1016/j.tranon.2024.102183, PMID: 39613524 PMC11629542

[102] Caggiano EG Hernandez AL Waldrop T Liu K Gatica-Gutierrez H Vargas-Hernández S . Mitochondrial responses to conventional and ultra-high dose rate (FLASH) radiation. bioRxiv: preprint server Biol. (2025). doi: 10.1101/2025.04.03.647049, PMID: 42448000

