# Correction: Influence factor and mechanism of FLASH effect

**DOI:** 10.3389/fonc.2025.1761396

**Published:** 2025-12-15

**Authors:** Tingyu Feng, Tianyang He, Wentao Ye, Lisha Xiang

**Affiliations:** 1 Division of Thoracic Tumor Multimodality Treatment and Department of Medical Oncology, Department of Radiation Oncology, Cancer Center, West China Hospital, Sichuan University, Chengdu, China; 2 West China School of Medicine, West China Hospital, Sichuan University, Chengdu, China

**Keywords:** ultra-high dose-rate, FLASH radiotherapy, mechanism, influencing factor, FLASH effect

Affiliation “^1^ Division of Thoracic Tumor Multimodality Treatment and Department of Medical Oncology, Department of Radiation Oncology, Cancer Center, West China Hospital, Sichuan University, Chengdu, China” was omitted from the paper. This affiliation has now been added for author “Tingyu Feng.”

Affiliation “^2^ West China School of Medicine, West China Hospital, Sichuan University, Chengdu, China” was erroneously given as “^1^ West China College of Clinical Medicine, Sichuan University, Chengdu, China.”

Affiliation “^1^Division of Thoracic Tumor Multimodality Treatment and Department of Medical Oncology, Department of Radiation Oncology, Cancer Center, West China Hospital, Sichuan University, Chengdu, China” was erroneously given as “^2^Division of Thoracic Tumor Multimodality Treatment and Department of Medical Oncology, Cancer Center, West China Hospital, Sichuan University, Chengdu, China.”

The original version of this article has been updated.

